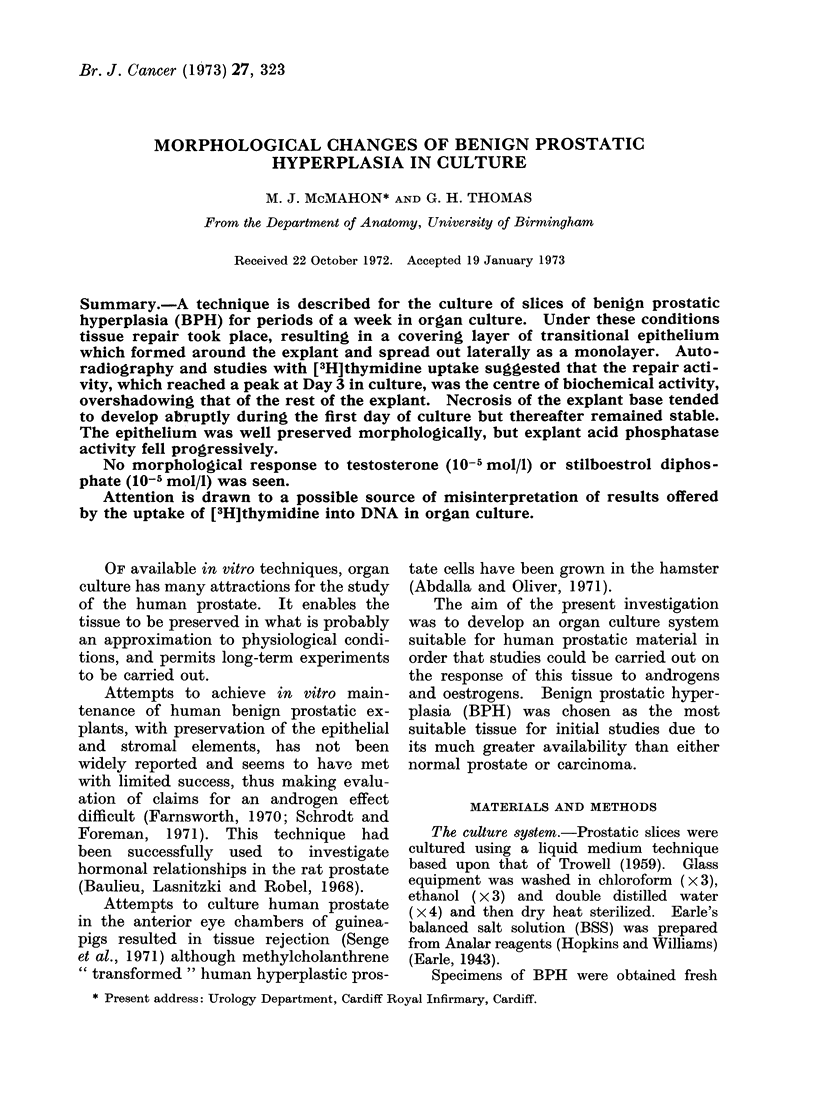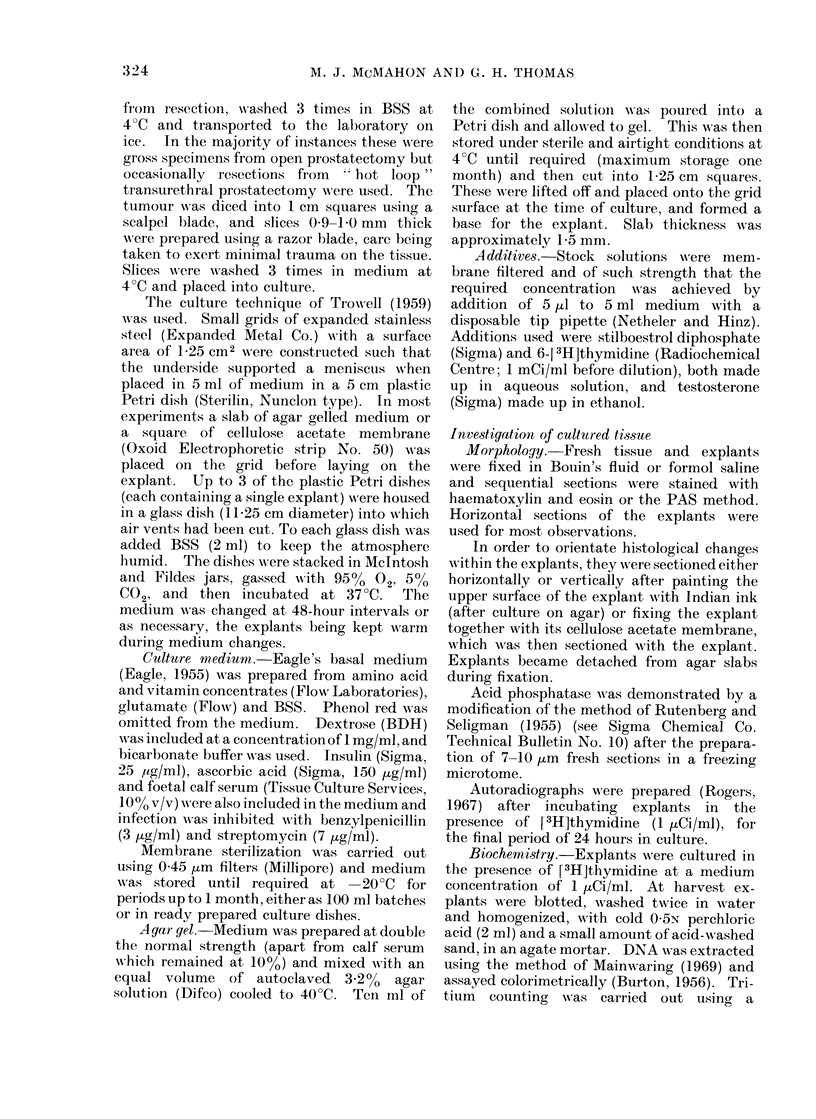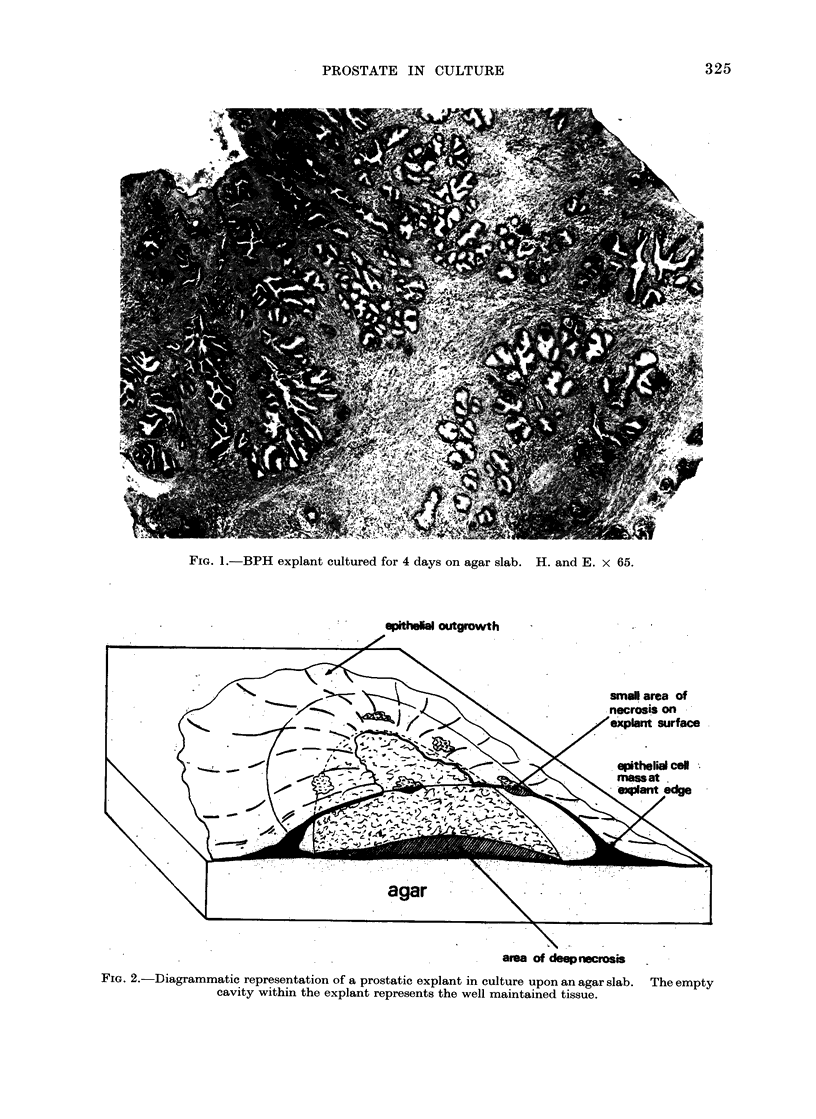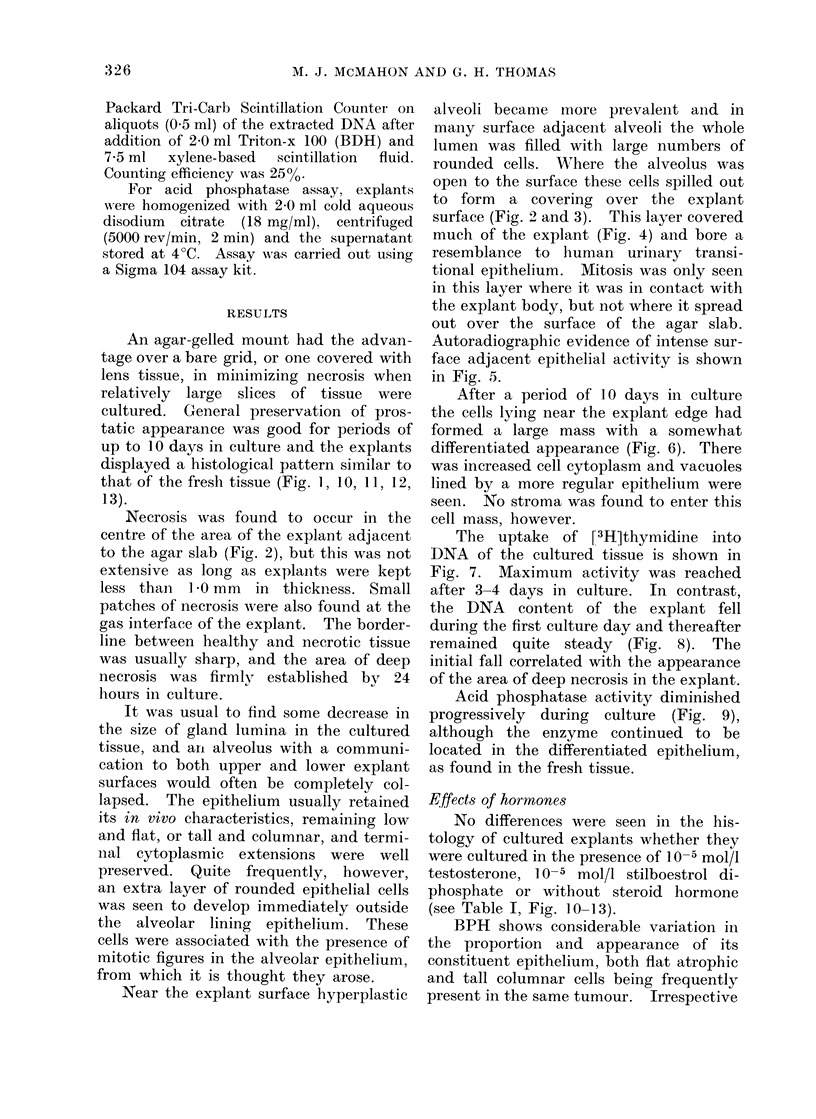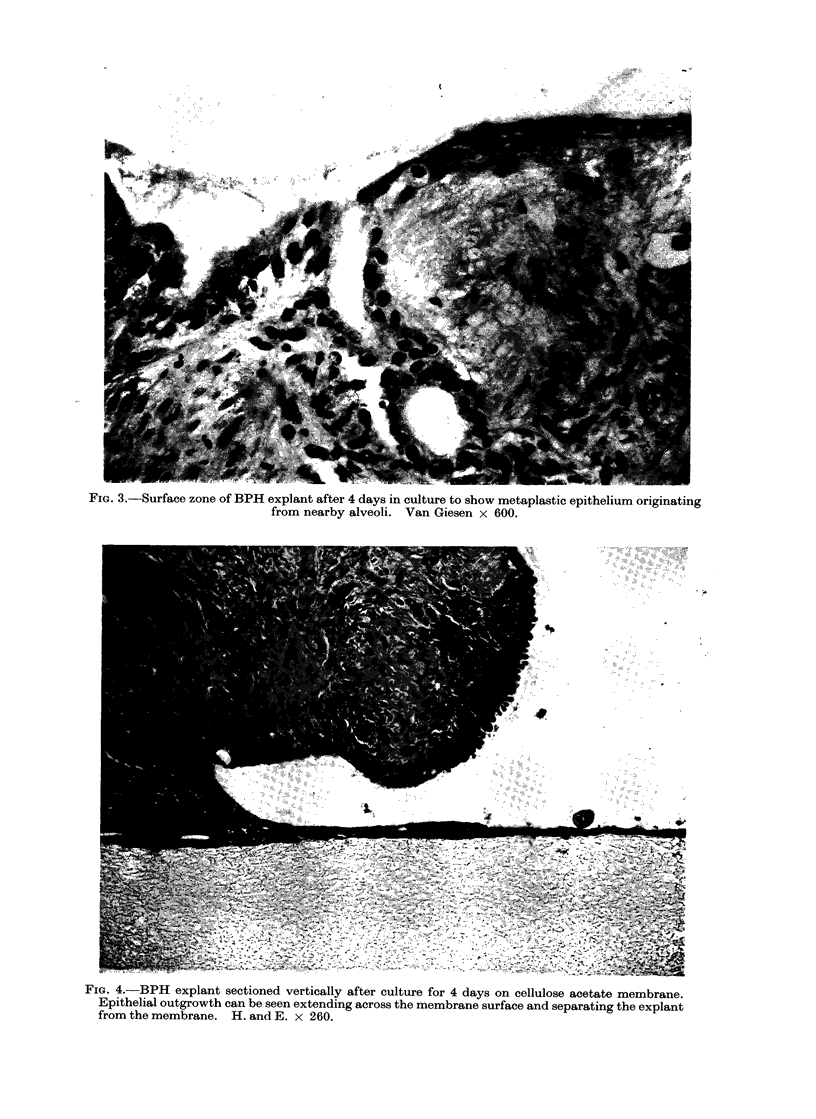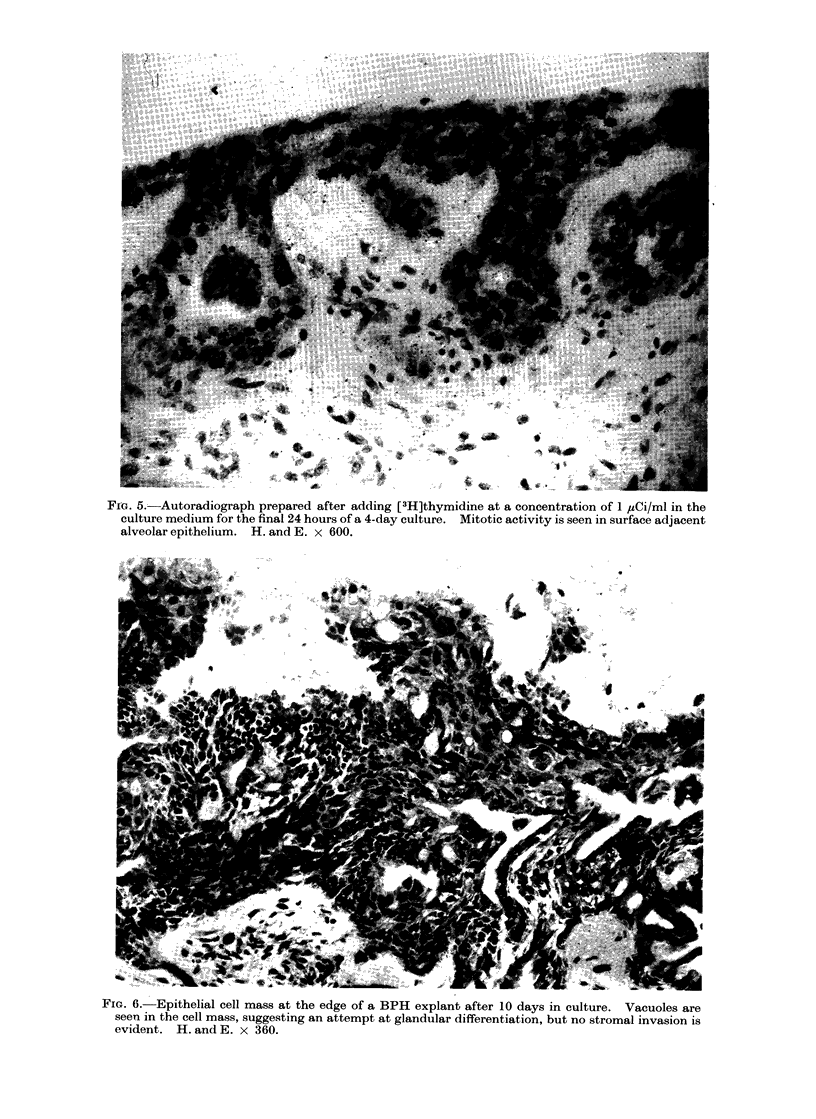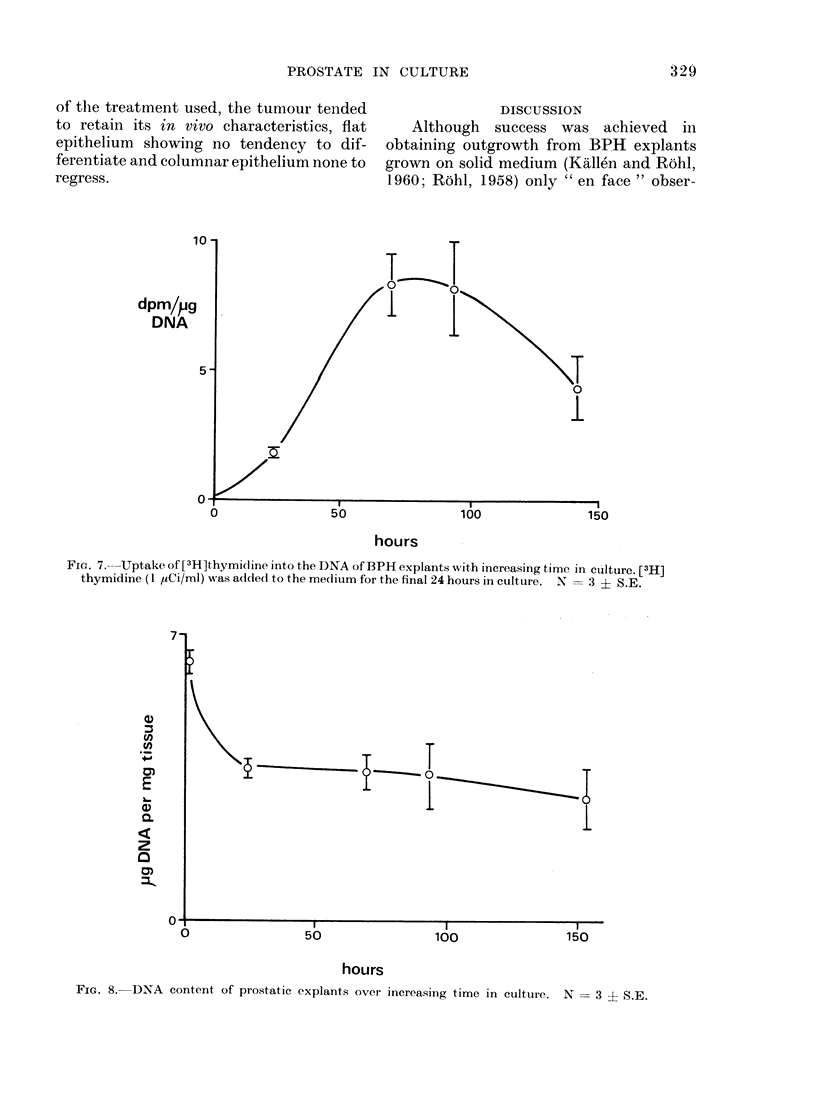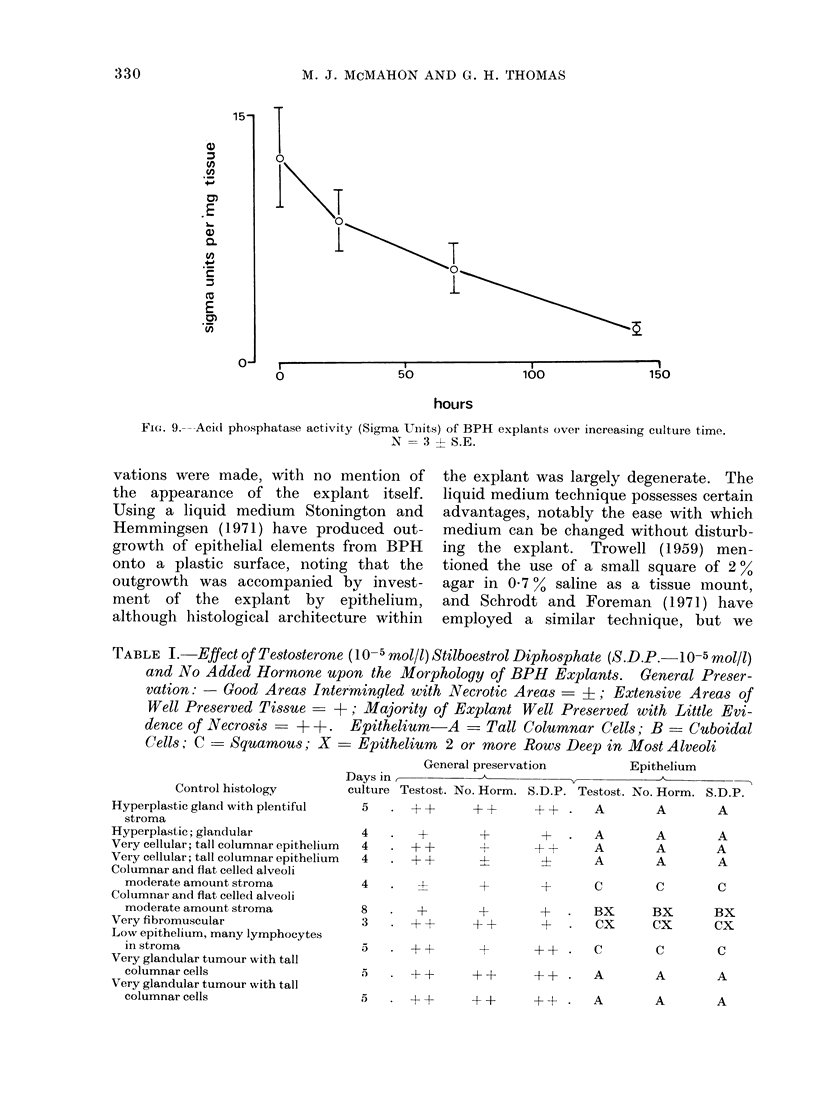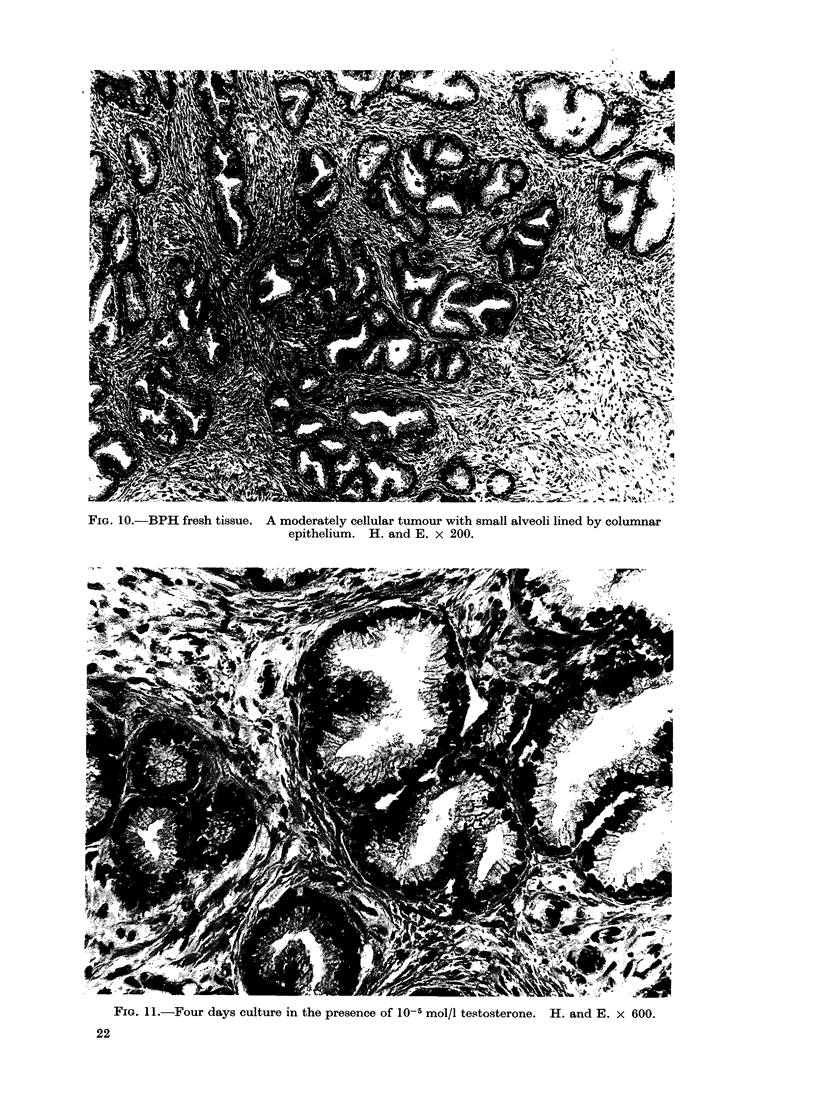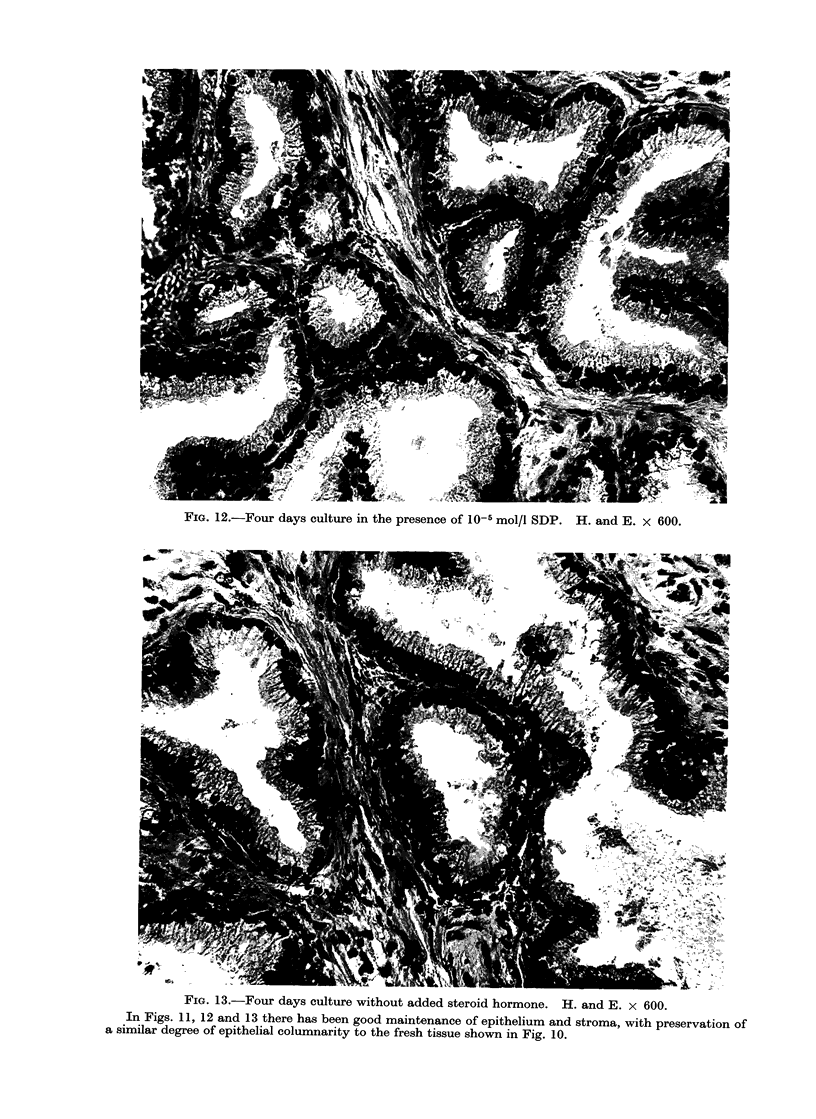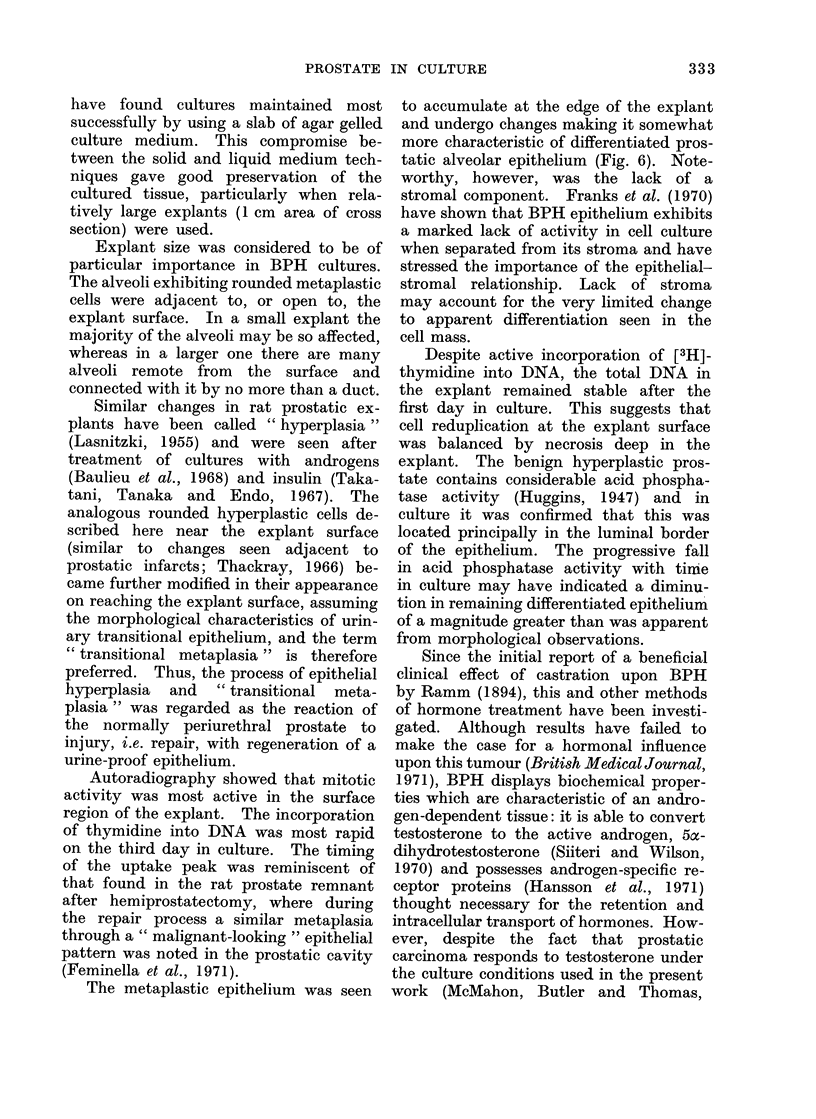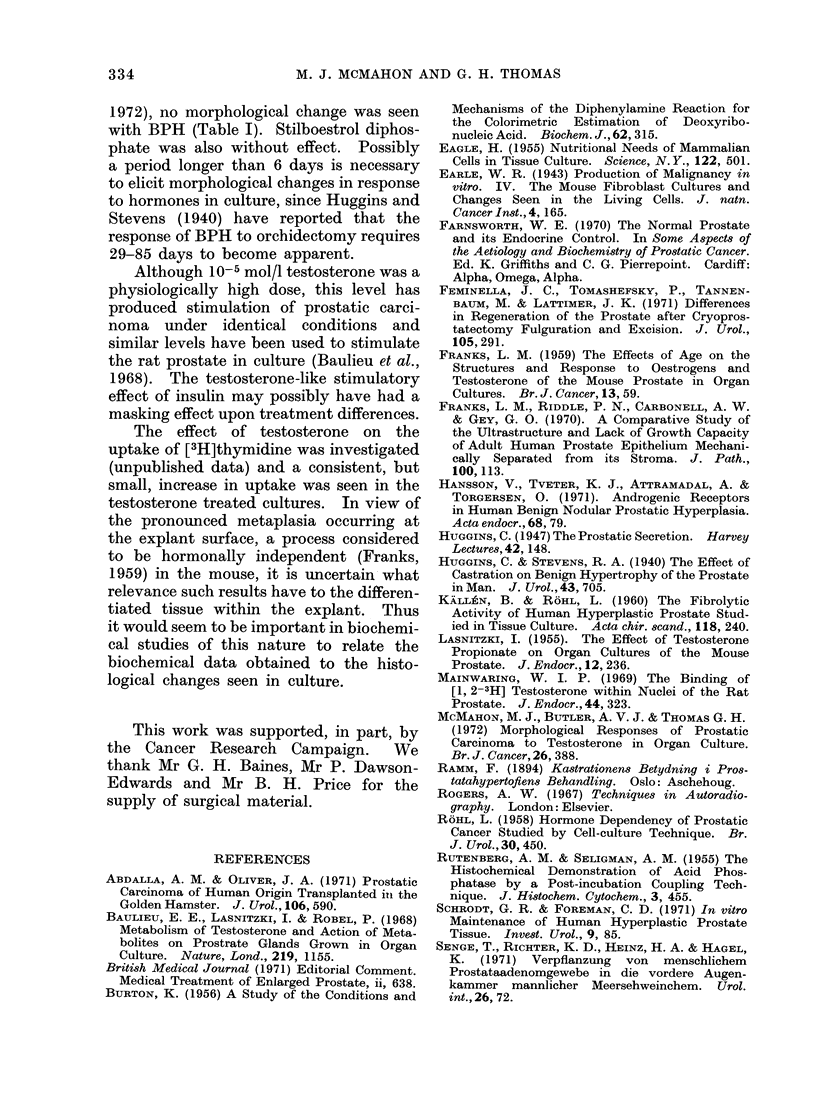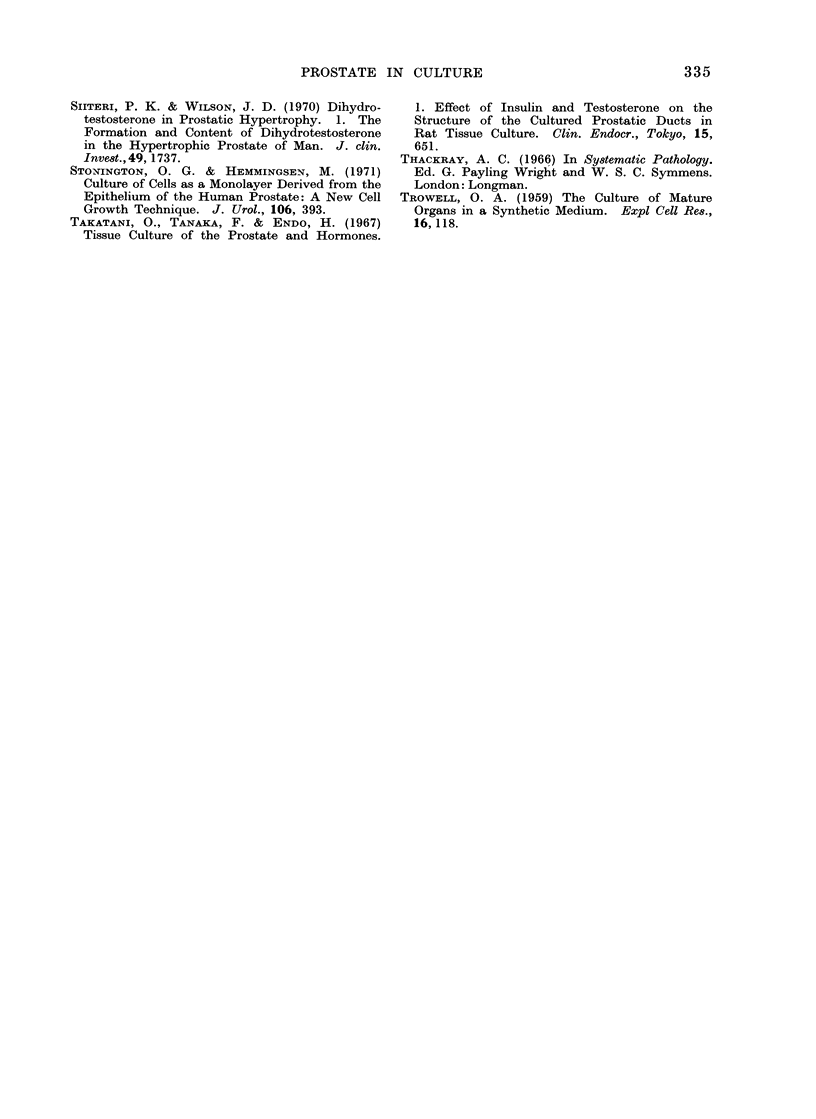# Morphological Changes of Benign Prostatic Hyperplasia in Culture

**DOI:** 10.1038/bjc.1973.39

**Published:** 1973-04

**Authors:** M. J. McMahon, G. H. Thomas

## Abstract

**Images:**


*Br. J. Cancer* (1973) **27**, 323

### MORPHOLOGICAL CHANGES OF BENIGN PROSTATIC HYPERPLASIA IN CULTURE

M. J. McMAHON* AND G. H. THOMAS

*From the Department of Anatomy, University of Birmingham*

Received 22 October 1972. Accepted 19 January 1973

**Summary.—A technique is described for the culture of slices of benign prostatic hyperplasia (BPH) for periods of a week in organ culture. Under these conditions tissue repair took place, resulting in a covering layer of transitional epithelium which formed around the explant and spread out laterally as a monolayer. Autoradiography and studies with [$^3$H]thymidine uptake suggested that the repair activity, which reached a peak at Day 3 in culture, was the centre of biochemical activity, overshadowing that of the rest of the explant. Necrosis of the explant base tended to develop abruptly during the first day of culture but thereafter remained stable. The epithelium was well preserved morphologically, but explant acid phosphatase activity fell progressively.**

**No morphological response to testosterone ($10^{-5}$ mol/l) or stilboestrol diphosphate ($10^{-5}$ mol/l) was seen.**

**Attention is drawn to a possible source of misinterpretation of results offered by the uptake of [$^3$H]thymidine into DNA in organ culture.**

OF available *in vitro* techniques, organ culture has many attractions for the study of the human prostate. It enables the tissue to be preserved in what is probably an approximation to physiological conditions, and permits long-term experiments to be carried out.

Attempts to achieve *in vitro* maintenance of human benign prostatic explants, with preservation of the epithelial and stromal elements, has not been widely reported and seems to have met with limited success, thus making evaluation of claims for an androgen effect difficult (Farnsworth, 1970; Schrodt and Foreman, 1971). This technique had been successfully used to investigate hormonal relationships in the rat prostate (Baulieu, Lasnitzki and Robel, 1968).

Attempts to culture human prostate in the anterior eye chambers of guinea-pigs resulted in tissue rejection (Senge *et al.*, 1971) although methylcholanthrene " transformed " human hyperplastic prostate cells have been grown in the hamster (Abdalla and Oliver, 1971).

The aim of the present investigation was to develop an organ culture system suitable for human prostatic material in order that studies could be carried out on the response of this tissue to androgens and oestrogens. Benign prostatic hyperplasia (BPH) was chosen as the most suitable tissue for initial studies due to its much greater availability than either normal prostate or carcinoma.

### MATERIALS AND METHODS

*The culture system.*—Prostatic slices were cultured using a liquid medium technique based upon that of Trowell (1959). Glass equipment was washed in chloroform (×3), ethanol (×3) and double distilled water (×4) and then dry heat sterilized. Earle's balanced salt solution (BSS) was prepared from Analar reagents (Hopkins and Williams) (Earle, 1943).

Specimens of BPH were obtained fresh

* Present address: Urology Department, Cardiff Royal Infirmary, Cardiff.

from resection, washed 3 times in BSS at 4°C and transported to the laboratory on ice. In the majority of instances these were gross specimens from open prostatectomy but occasionally resections from "hot loop" transurethral prostatectomy were used. The tumour was diced into 1 cm squares using a scalpel blade, and slices 0·9–1·0 mm thick were prepared using a razor blade, care being taken to exert minimal trauma on the tissue. Slices were washed 3 times in medium at 4°C and placed into culture.

The culture technique of Trowell (1959) was used. Small grids of expanded stainless steel (Expanded Metal Co.) with a surface area of 1·25 $cm^2$ were constructed such that the underside supported a meniscus when placed in 5 ml of medium in a 5 cm plastic Petri dish (Sterilin, Nunclon type). In most experiments a slab of agar gelled medium or a square of cellulose acetate membrane (Oxoid Electrophoretic strip No. 50) was placed on the grid before laying on the explant. Up to 3 of the plastic Petri dishes (each containing a single explant) were housed in a glass dish (11·25 cm diameter) into which air vents had been cut. To each glass dish was added BSS (2 ml) to keep the atmosphere humid. The dishes were stacked in McIntosh and Fildes jars, gassed with 95% $O_2$, 5% $CO_2$, and then incubated at 37°C. The medium was changed at 48-hour intervals or as necessary, the explants being kept warm during medium changes.

*Culture medium.*—Eagle's basal medium (Eagle, 1955) was prepared from amino acid and vitamin concentrates (Flow Laboratories), glutamate (Flow) and BSS. Phenol red was omitted from the medium. Dextrose (BDH) was included at a concentration of 1 mg/ml, and bicarbonate buffer was used. Insulin (Sigma, 25 $\mu$g/ml), ascorbic acid (Sigma, 150 $\mu$g/ml) and foetal calf serum (Tissue Culture Services, 10% v/v) were also included in the medium and infection was inhibited with benzylpenicillin (3 $\mu$g/ml) and streptomycin (7 $\mu$g/ml).

Membrane sterilization was carried out using 0·45 $\mu$m filters (Millipore) and medium was stored until required at −20°C for periods up to 1 month, either as 100 ml batches or in ready prepared culture dishes.

*Agar gel.*—Medium was prepared at double the normal strength (apart from calf serum which remained at 10%) and mixed with an equal volume of autoclaved 3·2% agar solution (Difco) cooled to 40°C. Ten ml of the combined solution was poured into a Petri dish and allowed to gel. This was then stored under sterile and airtight conditions at 4°C until required (maximum storage one month) and then cut into 1·25 cm squares. These were lifted off and placed onto the grid surface at the time of culture, and formed a base for the explant. Slab thickness was approximately 1·5 mm.

*Additives.*—Stock solutions were membrane filtered and of such strength that the required concentration was achieved by addition of 5 $\mu$l to 5 ml medium with a disposable tip pipette (Netheler and Hinz). Additions used were stilboestrol diphosphate (Sigma) and 6-[$^3$H]thymidine (Radiochemical Centre; 1 mCi/ml before dilution), both made up in aqueous solution, and testosterone (Sigma) made up in ethanol.

*Investigation of cultured tissue*

*Morphology.*—Fresh tissue and explants were fixed in Bouin's fluid or formol saline and sequential sections were stained with haematoxylin and eosin or the PAS method. Horizontal sections of the explants were used for most observations.

In order to orientate histological changes within the explants, they were sectioned either horizontally or vertically after painting the upper surface of the explant with Indian ink (after culture on agar) or fixing the explant together with its cellulose acetate membrane, which was then sectioned with the explant. Explants became detached from agar slabs during fixation.

Acid phosphatase was demonstrated by a modification of the method of Rutenberg and Seligman (1955) (see Sigma Chemical Co. Technical Bulletin No. 10) after the preparation of 7–10 $\mu$m fresh sections in a freezing microtome.

Autoradiographs were prepared (Rogers, 1967) after incubating explants in the presence of [$^3$H]thymidine (1 $\mu$Ci/ml), for the final period of 24 hours in culture.

*Biochemistry.*—Explants were cultured in the presence of [$^3$H]thymidine at a medium concentration of 1 $\mu$Ci/ml. At harvest explants were blotted, washed twice in water and homogenized, with cold 0·5N perchloric acid (2 ml) and a small amount of acid-washed sand, in an agate mortar. DNA was extracted using the method of Mainwaring (1969) and assayed colorimetrically (Burton, 1956). Tritium counting was carried out using a

FIG. 1.—BPH explant cultured for 4 days on agar slab. H. and E. × 65.

FIG. 2.—Diagrammatic representation of a prostatic explant in culture upon an agar slab. The empty cavity within the explant represents the well maintained tissue.

Packard Tri-Carb Scintillation Counter on aliquots (0·5 ml) of the extracted DNA after addition of 2·0 ml Triton-x 100 (BDH) and 7·5 ml xylene-based scintillation fluid. Counting efficiency was 25%.

For acid phosphatase assay, explants were homogenized with 2·0 ml cold aqueous disodium citrate (18 mg/ml), centrifuged (5000 rev/min, 2 min) and the supernatant stored at 4°C. Assay was carried out using a Sigma 104 assay kit.

### RESULTS

An agar-gelled mount had the advantage over a bare grid, or one covered with lens tissue, in minimizing necrosis when relatively large slices of tissue were cultured. General preservation of prostatic appearance was good for periods of up to 10 days in culture and the explants displayed a histological pattern similar to that of the fresh tissue (Fig. 1, 10, 11, 12, 13).

Necrosis was found to occur in the centre of the area of the explant adjacent to the agar slab (Fig. 2), but this was not extensive as long as explants were kept less than 1·0 mm in thickness. Small patches of necrosis were also found at the gas interface of the explant. The borderline between healthy and necrotic tissue was usually sharp, and the area of deep necrosis was firmly established by 24 hours in culture.

It was usual to find some decrease in the size of gland lumina in the cultured tissue, and an alveolus with a communication to both upper and lower explant surfaces would often be completely collapsed. The epithelium usually retained its *in vivo* characteristics, remaining low and flat, or tall and columnar, and terminal cytoplasmic extensions were well preserved. Quite frequently, however, an extra layer of rounded epithelial cells was seen to develop immediately outside the alveolar lining epithelium. These cells were associated with the presence of mitotic figures in the alveolar epithelium, from which it is thought they arose.

Near the explant surface hyperplastic alveoli became more prevalent and in many surface adjacent alveoli the whole lumen was filled with large numbers of rounded cells. Where the alveolus was open to the surface these cells spilled out to form a covering over the explant surface (Fig. 2 and 3). This layer covered much of the explant (Fig. 4) and bore a resemblance to human urinary transitional epithelium. Mitosis was only seen in this layer where it was in contact with the explant body, but not where it spread out over the surface of the agar slab. Autoradiographic evidence of intense surface adjacent epithelial activity is shown in Fig. 5.

After a period of 10 days in culture the cells lying near the explant edge had formed a large mass with a somewhat differentiated appearance (Fig. 6). There was increased cell cytoplasm and vacuoles lined by a more regular epithelium were seen. No stroma was found to enter this cell mass, however.

The uptake of [$^3$H]thymidine into DNA of the cultured tissue is shown in Fig. 7. Maximum activity was reached after 3–4 days in culture. In contrast, the DNA content of the explant fell during the first culture day and thereafter remained quite steady (Fig. 8). The initial fall correlated with the appearance of the area of deep necrosis in the explant.

Acid phosphatase activity diminished progressively during culture (Fig. 9), although the enzyme continued to be located in the differentiated epithelium, as found in the fresh tissue.

### *Effects of hormones*

No differences were seen in the histology of cultured explants whether they were cultured in the presence of $10^{-5}$ mol/l testosterone, $10^{-5}$ mol/l stilboestrol diphosphate or without steroid hormone (see Table I, Fig. 10–13).

BPH shows considerable variation in the proportion and appearance of its constituent epithelium, both flat atrophic and tall columnar cells being frequently present in the same tumour. Irrespective

FIG. 3.—Surface zone of BPH explant after 4 days in culture to show metaplastic epithelium originating from nearby alveoli. Van Giesen × 600.

FIG. 4.—BPH explant sectioned vertically after culture for 4 days on cellulose acetate membrane. Epithelial outgrowth can be seen extending across the membrane surface and separating the explant from the membrane. H. and E. × 260.

FIG. 5.—Autoradiograph prepared after adding [$^{3}$H]thymidine at a concentration of 1 $\mu$Ci/ml in the culture medium for the final 24 hours of a 4-day culture. Mitotic activity is seen in surface adjacent alveolar epithelium. H. and E. × 600.

FIG. 6.—Epithelial cell mass at the edge of a BPH explant after 10 days in culture. Vacuoles are seen in the cell mass, suggesting an attempt at glandular differentiation, but no stromal invasion is evident. H. and E. × 360.

of the treatment used, the tumour tended to retain its *in vivo* characteristics, flat epithelium showing no tendency to differentiate and columnar epithelium none to regress.

DISCUSSION

Although success was achieved in obtaining outgrowth from BPH explants grown on solid medium (Källén and Röhl, 1960; Röhl, 1958) only " en face " obser-

FIG. 7.—Uptake of [$^3$H]thymidine into the DNA of BPH explants with increasing time in culture. [$^3$H] thymidine (1 $\mu$Ci/ml) was added to the medium for the final 24 hours in culture. N = 3 $\pm$ S.E.

FIG. 8.—DNA content of prostatic explants over increasing time in culture. N = 3 $\pm$ S.E.

FIG. 9.—Acid phosphatase activity (Sigma Units) of BPH explants over increasing culture time. N = 3 ± S.E.

vations were made, with no mention of the appearance of the explant itself. Using a liquid medium Stonington and Hemmingsen (1971) have produced outgrowth of epithelial elements from BPH onto a plastic surface, noting that the outgrowth was accompanied by investment of the explant by epithelium, although histological architecture within the explant was largely degenerate. The liquid medium technique possesses certain advantages, notably the ease with which medium can be changed without disturbing the explant. Trowell (1959) mentioned the use of a small square of 2% agar in 0·7% saline as a tissue mount, and Schrodt and Foreman (1971) have employed a similar technique, but we

TABLE I.—*Effect of Testosterone* ($10^{-5}$ *mol/l*) *Stilboestrol Diphosphate* (*S.D.P.*—$10^{-5}$ *mol/l*) *and No Added Hormone upon the Morphology of BPH Explants. General Preservation: — Good Areas Intermingled with Necrotic Areas* = ±; *Extensive Areas of Well Preserved Tissue* = +; *Majority of Explant Well Preserved with Little Evidence of Necrosis* = ++. *Epithelium—A* = *Tall Columnar Cells; B* = *Cuboidal Cells;* C = *Squamous; X* = *Epithelium* 2 *or more Rows Deep in Most Alveoli*

| Control histology | Days in culture | General preservation | | | Epithelium | | |
|---|---|---|---|---|---|---|---|
| | | Testost. | No. Horm. | S.D.P. | Testost. | No. Horm. | S.D.P. |
| Hyperplastic gland with plentiful stroma | 5 | ++ | ++ | ++ | A | A | A |
| Hyperplastic; glandular | 4 | + | + | + | A | A | A |
| Very cellular; tall columnar epithelium | 4 | ++ | + | ++ | A | A | A |
| Very cellular; tall columnar epithelium | 4 | ++ | ± | ± | A | A | A |
| Columnar and flat celled alveoli moderate amount stroma | 4 | ± | + | + | C | C | C |
| Columnar and flat celled alveoli moderate amount stroma | 8 | + | + | + | BX | BX | BX |
| Very fibromuscular | 3 | ++ | ++ | + | CX | CX | CX |
| Low epithelium, many lymphocytes in stroma | 5 | ++ | + | ++ | C | C | C |
| Very glandular tumour with tall columnar cells | 5 | ++ | ++ | ++ | A | A | A |
| Very glandular tumour with tall columnar cells | 5 | ++ | ++ | ++ | A | A | A |

FIG. 10.—BPH fresh tissue. A moderately cellular tumour with small alveoli lined by columnar epithelium. H. and E. × 200.

FIG. 11.—Four days culture in the presence of $10^{-5}$ mol/l testosterone. H. and E. × 600.

FIG. 12.—Four days culture in the presence of $10^{-5}$ mol/l SDP. H. and E. × 600.

FIG. 13.—Four days culture without added steroid hormone. H. and E. × 600.

In Figs. 11, 12 and 13 there has been good maintenance of epithelium and stroma, with preservation of a similar degree of epithelial columnarity to the fresh tissue shown in Fig. 10.

have found cultures maintained most successfully by using a slab of agar gelled culture medium. This compromise between the solid and liquid medium techniques gave good preservation of the cultured tissue, particularly when relatively large explants (1 cm area of cross section) were used.

Explant size was considered to be of particular importance in BPH cultures. The alveoli exhibiting rounded metaplastic cells were adjacent to, or open to, the explant surface. In a small explant the majority of the alveoli may be so affected, whereas in a larger one there are many alveoli remote from the surface and connected with it by no more than a duct.

Similar changes in rat prostatic explants have been called " hyperplasia " (Lasnitzki, 1955) and were seen after treatment of cultures with androgens (Baulieu *et al.*, 1968) and insulin (Takatani, Tanaka and Endo, 1967). The analogous rounded hyperplastic cells described here near the explant surface (similar to changes seen adjacent to prostatic infarcts; Thackray, 1966) became further modified in their appearance on reaching the explant surface, assuming the morphological characteristics of urinary transitional epithelium, and the term " transitional metaplasia " is therefore preferred. Thus, the process of epithelial hyperplasia and " transitional metaplasia " was regarded as the reaction of the normally periurethral prostate to injury, *i.e.* repair, with regeneration of a urine-proof epithelium.

Autoradiography showed that mitotic activity was most active in the surface region of the explant. The incorporation of thymidine into DNA was most rapid on the third day in culture. The timing of the uptake peak was reminiscent of that found in the rat prostate remnant after hemiprostatectomy, where during the repair process a similar metaplasia through a " malignant-looking " epithelial pattern was noted in the prostatic cavity (Feminella *et al.*, 1971).

The metaplastic epithelium was seen to accumulate at the edge of the explant and undergo changes making it somewhat more characteristic of differentiated prostatic alveolar epithelium (Fig. 6). Noteworthy, however, was the lack of a stromal component. Franks *et al.* (1970) have shown that BPH epithelium exhibits a marked lack of activity in cell culture when separated from its stroma and have stressed the importance of the epithelial–stromal relationship. Lack of stroma may account for the very limited change to apparent differentiation seen in the cell mass.

Despite active incorporation of [$^3$H]-thymidine into DNA, the total DNA in the explant remained stable after the first day in culture. This suggests that cell reduplication at the explant surface was balanced by necrosis deep in the explant. The benign hyperplastic prostate contains considerable acid phosphatase activity (Huggins, 1947) and in culture it was confirmed that this was located principally in the luminal border of the epithelium. The progressive fall in acid phosphatase activity with time in culture may have indicated a diminution in remaining differentiated epithelium of a magnitude greater than was apparent from morphological observations.

Since the initial report of a beneficial clinical effect of castration upon BPH by Ramm (1894), this and other methods of hormone treatment have been investigated. Although results have failed to make the case for a hormonal influence upon this tumour (*British Medical Journal*, 1971), BPH displays biochemical properties which are characteristic of an androgen-dependent tissue: it is able to convert testosterone to the active androgen, 5$\alpha$-dihydrotestosterone (Siiteri and Wilson, 1970) and possesses androgen-specific receptor proteins (Hansson *et al.*, 1971) thought necessary for the retention and intracellular transport of hormones. However, despite the fact that prostatic carcinoma responds to testosterone under the culture conditions used in the present work (McMahon, Butler and Thomas,

1972), no morphological change was seen with BPH (Table I). Stilboestrol diphosphate was also without effect. Possibly a period longer than 6 days is necessary to elicit morphological changes in response to hormones in culture, since Huggins and Stevens (1940) have reported that the response of BPH to orchidectomy requires 29–85 days to become apparent.

Although $10^{-5}$ mol/l testosterone was a physiologically high dose, this level has produced stimulation of prostatic carcinoma under identical conditions and similar levels have been used to stimulate the rat prostate in culture (Baulieu *et al.*, 1968). The testosterone-like stimulatory effect of insulin may possibly have had a masking effect upon treatment differences.

The effect of testosterone on the uptake of [$^3$H]thymidine was investigated (unpublished data) and a consistent, but small, increase in uptake was seen in the testosterone treated cultures. In view of the pronounced metaplasia occurring at the explant surface, a process considered to be hormonally independent (Franks, 1959) in the mouse, it is uncertain what relevance such results have to the differentiated tissue within the explant. Thus it would seem to be important in biochemical studies of this nature to relate the biochemical data obtained to the histological changes seen in culture.

This work was supported, in part, by the Cancer Research Campaign. We thank Mr G. H. Baines, Mr P. Dawson-Edwards and Mr B. H. Price for the supply of surgical material.

### REFERENCES

ABDALLA, A. M. & OLIVER, J. A. (1971) Prostatic Carcinoma of Human Origin Transplanted in the Golden Hamster. *J. Urol.*, **106**, 590.

BAULIEU, E. E., LASNITZKI, I. & ROBEL, P. (1968) Metabolism of Testosterone and Action of Metabolites on Prostrate Glands Grown in Organ Culture. *Nature, Lond.*, **219**, 1155.

*British Medical Journal* (1971) Editorial Comment. Medical Treatment of Enlarged Prostate, ii, 638.

BURTON, K. (1956) A Study of the Conditions and Mechanisms of the Diphenylamine Reaction for the Colorimetric Estimation of Deoxyribonucleic Acid. *Biochem. J.*, **62**, 315.

EAGLE, H. (1955) Nutritional Needs of Mammalian Cells in Tissue Culture. *Science, N.Y.*, **122**, 501.

EARLE, W. R. (1943) Production of Malignancy *in vitro*. IV. The Mouse Fibroblast Cultures and Changes Seen in the Living Cells. *J. natn. Cancer Inst.*, **4**, 165.

FARNSWORTH, W. E. (1970) The Normal Prostate and its Endocrine Control. In *Some Aspects of the Aetiology and Biochemistry of Prostatic Cancer.* Ed. K. Griffiths and C. G. Pierrepoint. Cardiff: Alpha, Omega, Alpha.

FEMINELLA, J. C., TOMASHEFSKY, P., TANNENBAUM, M. & LATTIMER, J. K. (1971) Differences in Regeneration of the Prostate after Cryoprostatectomy Fulguration and Excision. *J. Urol.*, **105**, 291.

FRANKS, L. M. (1959) The Effects of Age on the Structures and Response to Oestrogens and Testosterone of the Mouse Prostate in Organ Cultures. *Br. J. Cancer*, **13**, 59.

FRANKS, L. M., RIDDLE, P. N., CARBONELL, A. W. & GEY, G. O. (1970). A Comparative Study of the Ultrastructure and Lack of Growth Capacity of Adult Human Prostate Epithelium Mechanically Separated from its Stroma. *J. Path.*, **100**, 113.

HANSSON, V., TVETER, K. J., ATTRAMADAL, A. & TORGERSEN, O. (1971). Androgenic Receptors in Human Benign Nodular Prostatic Hyperplasia. *Acta endocr.*, **68**, 79.

HUGGINS, C. (1947) The Prostatic Secretion. *Harvey Lectures*, **42**, 148.

HUGGINS, C. & STEVENS, R. A. (1940) The Effect of Castration on Benign Hypertrophy of the Prostate in Man. *J. Urol.*, **43**, 705.

KÄLLÉN, B. & RÖHL, L. (1960) The Fibrolytic Activity of Human Hyperplastic Prostate Studied in Tissue Culture. *Acta chir. scand.*, **118**, 240.

LASNITZKI, I. (1955). The Effect of Testosterone Propionate on Organ Cultures of the Mouse Prostate. *J. Endocr.*, **12**, 236.

MAINWARING, W. I. P. (1969) The Binding of [1, 2-$^3$H] Testosterone within Nuclei of the Rat Prostate. *J. Endocr.*, **44**, 323.

MCMAHON, M. J., BUTLER, A. V. J. & THOMAS G. H. (1972) Morphological Responses of Prostatic Carcinoma to Testosterone in Organ Culture. *Br. J. Cancer*, **26**, 388.

RAMM, F. (1894) *Kastrationens Betydning i Prostatahypertofiens Behandling.* Oslo: Aschehoug.

ROGERS, A. W. (1967) *Techniques in Autoradiography.* London: Elsevier.

RÖHL, L. (1958) Hormone Dependency of Prostatic Cancer Studied by Cell-culture Technique. *Br. J. Urol.*, **30**, 450.

RUTENBERG, A. M. & SELIGMAN, A. M. (1955) The Histochemical Demonstration of Acid Phosphatase by a Post-incubation Coupling Technique. *J. Histochem. Cytochem.*, **3**, 455.

SCHRODT, G. R. & FOREMAN, C. D. (1971) *In vitro* Maintenance of Human Hyperplastic Prostate Tissue. *Invest. Urol.*, **9**, 85.

SENGE, T., RICHTER, K. D., HEINZ, H. A. & HAGEL, K. (1971) Verpflanzung von menschlichem Prostataadenomgewebe in die vordere Augenkammer mannlicher Meersehweinchem. *Urol. int.*, **26**, 72.

SIITERI, P. K. & WILSON, J. D. (1970) Dihydrotestosterone in Prostatic Hypertrophy. 1. The Formation and Content of Dihydrotestosterone in the Hypertrophic Prostate of Man. *J. clin. Invest.*, **49**, 1737.

STONINGTON, O. G. & HEMMINGSEN, M. (1971) Culture of Cells as a Monolayer Derived from the Epithelium of the Human Prostate: A New Cell Growth Technique. *J. Urol.*, **106**, 393.

TAKATANI, O., TANAKA, F. & ENDO, H. (1967) Tissue Culture of the Prostate and Hormones. 1. Effect of Insulin and Testosterone on the Structure of the Cultured Prostatic Ducts in Rat Tissue Culture. *Clin. Endocr., Tokyo*, **15**, 651.

THACKRAY, A. C. (1966) In *Systematic Pathology*. Ed. G. Payling Wright and W. S. C. Symmens. London: Longman.

TROWELL, O. A. (1959) The Culture of Mature Organs in a Synthetic Medium. *Expl Cell Res.*, **16**, 118.